# Health literacy in asthma and chronic obstructive pulmonary disease (COPD) care: a narrative review and future directions

**DOI:** 10.1186/s12931-022-02290-5

**Published:** 2022-12-19

**Authors:** Iraj Poureslami, J. Mark FitzGerald, Noah Tregobov, Roger S. Goldstein, M. Diane Lougheed, Samir Gupta

**Affiliations:** 1 Division of Respiratory Medicine, Centre for Lung Health, Vancouver Coastal Health Research Institute, University of British Columbia, 716-828 West 10th Avenue, Vancouver, BC V5Z 1M9 Canada; 2 Canadian Multicultural Health Promotion Society (CMHPS), Vancouver, BC Canada; 3 Faculty of Medicine, Vancouver-Fraser Medical Program, University of British Columbia, Vancouver, BC Canada; 4 Department of Physical Therapy, Temerty Faculty of Medicine, University of Toronto, Toronto, Canada; 5 Respiratory Medicine, Westpark Healthcare Centre, Toronto, Canada; 6 Department of Medicine, Temerty Faculty of Medicine, University of Toronto, Toronto, ON Canada; 7 Asthma Research Unit, Department of Medicine, Kingston Health Sciences Centre, Queen’s University, Kingston, ON Canada; 8 Institute for Clinical Evaluative Sciences, Toronto, ON Canada; 9 Unity Health, Li Ka Shing Knowledge Institute of St. Michael’s Hospital, Toronto, ON Canada; 10 Division of Respirology, Department of Medicine, University of Toronto, Toronto, ON Canada

**Keywords:** Chronic airway disease, Health literacy, Cultural competence, Patient-centered care, Narrative review

## Abstract

Respiratory self-care places considerable demands on patients with chronic airways disease (AD), as they must obtain, understand and apply information required to follow their complex treatment plans. If clinical and lifestyle information overwhelms patients’ HL capacities, it reduces their ability to self-manage. This review outlines important societal, individual, and healthcare system factors that influence disease management and outcomes among patients with asthma and chronic obstructive pulmonary disease (COPD)—the two most common ADs. For this review, we undertook a comprehensive literature search, conducted reference list searches from prior HL-related publications, and added insights from international researchers and scientists with an interest in HL. We identified methodological limitations in currently available HL measurement tools in respiratory care. We also summarized the issues contributing to low HL and system-level cultural incompetency that continue to be under-recognized in AD management and contribute to suboptimal patient outcomes. Given that impaired HL is not commonly recognized as an important factor in AD care, we propose a three-level patient-centered model (strategies) designed to integrate HL considerations, with the goal of enabling health systems to enhance service delivery to meet the needs of all AD patients.

## Background

As the prevalence of chronic diseases continues to increase, along with their burden on health systems and patients [1, 2], there is an increasing awareness that patients will benefit from being empowered to actively engage in disease self-management [3, 4]. This has led to patient-centered care models [5–7], which include collaboration between patients and their healthcare providers, and enhanced respect for patient values, preferences and expressed needs [5]. Although a patient-centered approach relies on improving patients’ disease-related knowledge through educational interventions [6, 7], knowledge alone may not sufficiently motivate or enable patients to become active participants in self-management [8, 9]. Patient engagement can be hindered by many factors, including difficulty navigating the healthcare system, misunderstanding information, non-adherence to instructions, and lack of regular, ongoing provider contacts [10–12]. Health literacy (HL) has increasingly become recognized as both a cause of and a solution to this problem, as it is a determinant of patient empowerment [13, 14] and disease management success [8, 15–17]. Studies among patients with diabetes, cancer, arthritis, cardiovascular disease, and stroke have all shown associations between low HL and worse health outcomes [18–20]. Unfortunately, despite the importance of HL in self-management of chronic airway diseases (ADs) such as asthma and chronic obstructive pulmonary disease (COPD), its application in empowering AD patients to make informed decisions about their health remains limited [11, 16, 21].

Herein, we describe a model for respiratory patient-centered care that is culturally and HL-competent and explore the potential impact of these competencies on care delivery, individuals, and communities. Our goal was to provide a framework and practical approaches that can be applied to improve patient-centered care through HL. To achieve this, we applied insights from the literature and our own practical experiences (including work with national and international HL-focused groups) [22, 23] to suggest strategies to integrate of HL and cultural competency at a system level.

### Overview of health literacy

In 2000, Ratzan and Parker [24] defined HL as: “*The degree to which individuals have the capacity to obtain, process, and understand basic health information and services needed to make appropriate health decisions.*” The Canadian Expert Panel on Health Literacy (CEPHL) [25] and the Calgary Charter on Health Literacy [26] developed a model of HL which included five main domains (Table 1) and defined HL as a person’s *capability to obtain, understand, communicate, evaluate, and use health information to make appropriate health-related decisions*”. The importance of HL for each of these domains has been well established individually [27, 28], and the “five-domain model of HL” has been endorsed and approved by different HL researchers and experts, as essential skills that a person may require to effectively navigate and obtain health information and care services related to their health issues [29, 30]. In addition, an individual’s ability to understand and calculate numerical information (“numeracy”) (Table 1) [31] is a necessary skill for an individual to understand and apply information provided in the health care system. Historically, researchers have considered numeracy to be a HL skill individually [32], however, since numeracy is a variable that is applicable to all core 5 HL domains [33], many researchers assess it across the domains rather than independently [33, 34]. HL is now considered a major determinant of overall health [27, 35–38], and an essential *life skill* [37, 39–41]. HL is also viewed through a *population health* lens, as health literate individuals improve the overall health of a community [42, 43]; and as a component of *social capital*, with low HL contributing to health inequalities [44]. Finally, low HL is associated with increased health care costs [45, 46].

**Table 1:** Health literacy & Numeracy domains definition/example Adopted from: Shum J, Poureslami I, Wiebe D, et al. Airway diseases and health literacy (HL) measurement tools: A systematic review to inform respiratory research and practice. Patient Educ Couns. 2018; 101(4): 596–618

***Access*** Being able to navigate and find health information—it is more than the availability of information and services. It is mediated by education, culture, and language, by the communication skills of professionals, by the nature of materials and messages, and by the settings in which health-related supports are provided e.g., “I have the skills to find the health information I want.”
***Understand*** Knowledge about a subject or situation and comprehension of the health condition and information—Cambridge Dictionaries e.g., “How confident do you feel you are able to follow the instructions on the label of your inhaler”?
***Evaluate*** To be able to determine whether information/service is applicable to self—to judge or calculate the quality, importance, truthfulness, or value of information—Cambridge Dictionaries e.g., “I have the skills to judge which health information can be trusted”
***Communicate*** To share information with others (doctor, caregiver, family members, etc.) by speaking, writing, and body language—Cambridge Dictionaries e.g., “I have the skills to describe my health concerns to others”
***Use*** Adapting and applying information to daily life for disease management—to take, hold, or deploy information as a means of accomplishing or achieving health outcomes—Oxford Dictionaries e.g., “I can use the information received from doctor/hospital to set my disease management goal”
***Health numeracy*** The degree to which individuals have the capacity to access, process, interpret, communicate, and act on numerical, quantitative, and graphical health information needed to make effective health decisions e.g. “I can understand numerical information in my medication/treatment instructions and apply it in my disease management”

**Table 2:** a. Studies Evaluating Clinical Outcomes that Reported One or More HL Outcomes. b. Studies Evaluating Behavioral Outcomes that Reported One or More HL Outcomes. c. Studies Evaluating Social/Psychological Outcomes that Reported One or More HL Outcomes

a							
Study	Design (Sample size)	Population/Disease type/Setting	Age/Sex	Tools applied to measure clinical outcomes of interest	Intervention	Main outcome measured	Key findings related to health literacy outcomes
Apter AJ, Wang X, Bogen DK, Rand CS, McElligott S, Polsky D, et al. Problem solving to improve adherence and asthma outcomes in urban adults with moderate or severe asthma: A randomized controlled trial. J Allergy Clin Immunol. 2011; 128:516–23.e1. 10.1016/j.jaci.2011.05.010	RCT^π^ (333)	Moderate or severe Asthma. Inpatients at large inner-city hospital and specialty respiratory clinic in US	49 ± 14 years 72% female	S-TOFHLA^1^ ACT^2^, Health-related Quality of Life (HRQL)^3^, FEV1, and disease knowledge	Problem-solving (PS) approach vs standard asthma education (AE) Checked Reading ability and word Understanding	**Treatment adherence and Healthcare services utilization**	Mean treatment adherence (61% ± 27%) declined significantly (p = 0.0004) over time by 14% and 10% in the intervention and control groups, respectively. Asthma control improved overall by 15% (p = 0.002). Problem-solving (intervention) was not better than asthma education (control) in improving disease knowledge and asthma outcomes, and reducing hospitalizations and ED visits (all p > .05)
Azkan Ture D, Bhattacharya S, Demirci H and Yildiz T. Health Literacy and Health Outcomes in Chronic Obstructive Pulmonary Disease Patients: An Explorative Study. Front. Public Health. 2022; 10:846768. https://doi.org/10.3389/fpubh.2022.846768	Pragmatic intervention study (336)	Moderate to Severe COPD in Turkey	62.5 ± 10.04 years 85% Male	HLS-EU^4^, CRQ^5^, MMRC^6^	Health coaching study Included: knowledge, understanding, and Use aspect of HL	**Disease severity**	The results shown the proportion of patients with inadequate HL was higher in the severe COPD group (73.20%). In patients with inadequate HL, the risk of developing severe COPD was 1.80 times higher
Eikelenboom N, van Lieshout J, Jacobs A, et al. Effectiveness of personalised support for self-management in primary care: a cluster randomised controlled trial. Br J Gen Pract. 2016;66(646): e354–e361. https://doi.org/10.3399/bjgp16X684985	Cluster RCT (644)	Adult patients with at least one chronic condition (asthma, COPD, diabetes mellitus, or cardiovascular Diseases in Netherland	65.8 ± 10.5 years 53% Male	S-TOFHLA	Personalized self-management support training sessions Checked Reading ability and word Understanding	**Patients’ activation and health-related behaviours**	Personalized self-management intervention (training sessions) has not effect on Patient Activation (score did not differ significantly between the control and intervention groups at 6 months and follow-up assessments, but, the effect of the intervention was significant on the patients performed self-monitoring (intervention group scored higher than the control group (p = 0.01). The HL scores correlated significantly with outcomes of interest (p < 0.001), but the differences were not significant between the study groups
Thom DH, Willard-Grace R, Tsao S, Hessler D, Huang B, DeVore D, et al. Randomized Controlled Trial of Health Coaching for Vulnerable Patients with Chronic Obstructive Pulmonary Disease. Annals ATS. 208; 15 (10): 1159–1168	RCT (192)	Moderate to severe COPD. Patients in seven urban public health primary care clinics in US	61.3 ± 7.6 years 65.5% male	CRQ, Chronic Illness Care, and Patient Health Outcomes, HRQL, Healthcare utilization	Health coaching training vs usual care Included Communication, Understanding, and Use aspect of HL	**ED Visits, Hospitalization rates, and HRQL**	There were no significant differences between the intervention (health coaching) and control (usual care) groups in either primary or secondary outcomes (p > .05). The findings from this study may inform expectations of benefits and limitations of health coaching for patients with COPD
Apter AJ, Bryant-Stephens T, Morales Andrea J, Wan F, Hardy S, Reed-Wells S, et al. Using IT to improve access, communication, and asthma in African American and Hispanic/Latino Adults: Rationale, design, and methods of a randomized controlled trial. Contemporary Clinical Trials. 2015; 44: 119–128 https://doi.org/10.1016/j.cct.2015.08.001	RCT (301)	Moderate to severe Asthma. Patients in 1 University Medical Complex in the US	49 ± 12 87% female	S-TOFHLA, eHEALS^5^, ANQ^6^. ACT, Asthma Control, HRQL, assessed literacy and language barriers, Healthcare utilization	Patient portal training (PT) vs patient portal training plus home visits (PT + HV) Checked Reading ability and word Understanding	**Asthma Control, Health-related Quality of Life, patient–** **provider communication, and health outcomes**	Both the PT and PT + HV groups improved, with fewer asthma symptoms, better QOL, less need for oral steroids, fewer asthma-related hospitalizations and ED visits per year. In all measures of effect, the group receiving home visits showed more improvement, but in hospitalization, the improvement was not statistically significant (P > 0.05). This trial improved communication with healthcare providers in a population of high asthma morbidity and low-income through the use of electronic patient portals and home visits
Wang LH, Zhao Y, Chen LY, Zhang L, Zhang YM. The effect of a nurse-led self-management program on outcomes of patients with chronic obstructive pulmonary disease. Clin. Respir. J. 2019; https://doi.org/10.1111/crj.13112	RCT (154)	Moderate to severe COPD. Patients in a teaching hospital in China	68.7 ± 6.2 76.6% male	SGRQ^7^, 6MWD^8^, and CTCPSQ^9^, Disease satisfaction, exercise capacity, hospital readmission (open-ended questionnaire)	Nurse-led self-management program in addition to routine care vs usual care Included Communication, Understanding, and Use aspects of HL	**Hospital readmission, emergency department visits, Exercise tolerance, HRQL, satisfaction**	The Intervention group showed significantly fewer COPD-related hospital admissions (p = 0.03) and emergency department visits (p = 0.001) compared to the control group. Intervention group participants had greater satisfaction with health care and higher quality of life (both p = 0.001), compared to the control group. The intervention group also had significantly greater improvement in exercise capacity and health status (p < 0.05) compared with control participants The Nurse-led self-management program was effective in improving COPD patient knowledge and disease management skills to manage symptoms and exacerbation
Monninkhof E, van der Valk P, van der Palen J, van Herwaarden C, Zielhuis G. Effects of a comprehensive self-management programme in patients with chronic obstructive pulmonary disease. Eur Respir J. 2003; 22(5): 815–20	RCT (248)	Moderately severe COPD. Outpatient pulmonary clinics in the Netherlands	65 ± 7 years 68.15% male	SGRQ and HRQL, Exercise capacity, Self-confidence, and health outcomes	Comprehensive self-management intervention vs. standard of care Included Understanding, and Use aspects of HL	**Exacerbation rates, HRQL, physical activity**	No statistically significant differences were observed between the Intervention and control groups over 1 year (p > 0.05). No significant differences in symptom scores and 6-min walking distance were found within and between groups (p > 0.05). The intervention group reported more exacerbations than the control group. This study did not show positive effects of a self-management program among moderately severe chronic obstructive pulmonary disease patients
Goeman D, Jenkins C, Crane M, Paul E, Douglass J. Educational intervention for older people with asthma: A randomised controlled trial,. Patient Education and Counseling. 2013; 93 (3): 586–595, ISSN 0738–3991, https://doi.org/10.1016/j.pec.2013.08.014	RCT (123)	Asthma. Outpatients from emergency departments, GP clinics and pharmacies in Australia	67.4 ± 6.4 72.4% female	ACT, HROL, Patient Health Questionnaire, and MAQ^10^	Person-centered self-management education intervention vs. written (brochure) information-only education Included Understanding, and Use aspects of HL	**Asthma Control and Exacerbation rates**	Intervention group participants experienced significant improvements in asthma control and quality of life, (both p < 0.01). Adherence to asthma preventer medication improved in both study groups with no statistically significant difference (p = 0.17. The exacerbation rates in both study groups decreased during the study period and the difference between groups was not statistically significant (p = 0.52). Asthma outcomes in older individuals may be improved by delivering tailored education that identifies specific patient concerns and unmet needs
Ko FW,Cheung NK, Rainer TH, Lum C, Wong I, Hu DS. Comprehensive care programme for patients with chronic obstructive pulmonary disease: a randomised controlled. Thorax 2017;72:122–128. https://doi.org/10.1136/thoraxjnl-2016-208396.trial.	RCT (180)	COPD Patients discharged from hospital after having acute exacerbation. Department of pulmonary medicine of a teaching hospital in Netherlands	74.7 ± 8.2 95.6% male	MMRC, 6MWD, SGRQ, HRQL, exercise capability. Health outcomes	Comprehensive educational program vs. usual care Included Communication, Understanding, and Use aspects of HL	**Hospital Readmission and length of hospital stay**	The incident rate of readmission in the intervention group was significantly lower than the control group (p = 0.047) compared with control group participants. The intervention patients had shorter length of hospital stay for acute exacerbation than the control group (p ≤ 0.001). There were no improvements in 6-min walk test, MMRC score and SGRQ score at the 12-month follow-up, compared to baseline, in either group A comprehensive, individualized care plan could decrease the hospital readmission rate and length of hospital stay compared with usual care. However, the intervention had minimal effects on self-efficacy and health-promoting behaviour
Mayo PH, Richman J, Harris HW. Results of a program to reduce admissions for adult asthma. Ann Intern Med. 1990 Jun 1;112(11):864–71. https://doi.org/10.7326/0003-4819-112-11-864. PMID: 2344111	RCT (104)	Moderate to severe Asthma with multiple hospitalizations for asthma attacks Patients in a general hospital in the US	42.7 ± 13.3 72.12% female	Self-reported checklist of self-treatment (using ICS with spacer and taking prednisone based on pattern of asthma symptoms and exacerbation, and applying peak fellow meter daily), inhaler techniques, and disease knowledge	Intensive personalized self-management education intervention plus special clinic treatment vs. routine clinic care Included Communication, Understanding, Evaluation, and Use aspects of HL	**Hospital readmissions and hospital days used**	The intervention resulted in a threefold reduction in readmission rate and a twofold reduction in hospital days use rate (p < 0.003 and p < 0.004, respectively) in the intervention group compared to the control group The educational intervention reduced hospital use among a group of adults with asthma, but it did not influence patient’s decision to use provided training to initiate self-treatment for asthma exacerbation
Fan VS, Gaziano JM, Lew R, Bourbeau J, Adams SG, Leatherman S, Thwin SS, Huang GD, Robbins R, Sriram PS, et al. A comprehensive care management program to prevent chronic obstructive pulmonary disease hospitalizations: a randomized, controlled trial. Ann Intern Med 2012;156:673–683	RCT (426)	Severe COPD. 20 Veterans Affairs hospital-based outpatient clinics across the US	65.9 ± 8.4 96% male	SGRQ, PHQ^11^, CCQ12, COPD exacerbations and hospitalizations, HRQL, patient satisfaction, disease knowledge, and self-efficacy	Comprehensive care management educational intervention vs. usual care Included Understanding, Communication, and Use aspects of HL	**COPD hospitalization, exacerbation,** **Mortality**	COPD-related hospitalization decreased in both study groups with no statistically significant difference between the groups (p = 0.62). No significant difference was found between two groups for treating exacerbation (p = 0.118). Significantly more deaths due to COPD was reported in the intervention group compared to the usual care group (p = 0.003). Significant improvement was observed in self-confidence in managing COPD within the intervention group (p = 0.044) The intervention had no effect in reduced COPD-related hospitalizations, exacerbation, or COPD knowledge. The intervention did not improve COPD knowledge, nor did it yield a structural behavioral change to motivate intervention patients to use their action plan for worsening respiratory symptoms. Behaviour change requires both a sufficient understanding of the problem and the self-confidence to address it effectively
Galbreath AD, Smith B, Wood PR, et al. Assessing the value of disease management: impact of 2 disease management strategies in an underserved asthma population. Ann Allergy Asthma Immunol. 2008;101:599–607	RCT (902)	Moderate to severe Asthma. Patients from one general hospital in the US (429 adults; 473 children)	Adult: 42.7 ± 12.3 77.6% female Pediatric: 9.47 ± 3.3 59.5% male	HLQL, ATC, ATAQ^13^, and MAQ	Telephonic Asthma management (TAM) consulting, Augmented approach plus in-home visits by a respiratory therapist-AAM), vs. usual care Included Communication, Understanding, and Use aspects of HL	**Exacerbations, health care utilization, HRQL**	No significant differences found between study groups in time to exacerbation event or health care utilization (p > 0.05) for either pediatric or adult patients. TAM group in adult group had greater improvement in HRQL (p = .04) and a decrease in asthma symptoms (p = .001). Compared to usual care, the interventions did not result in significant improvement in disease knowledge, nor did it yield a gain in skills to improve clinical outcomes in either adults or children
Khdour MR, Kidney JC, Smyth BM, McElnay JC. Clinical pharmacy-led disease and medicine management programme for patients with COPD. British Journal of Clinical Pharmacology. 2009; 68, 588–598. https://doi.org/10.1111/J.1365-2125.2009.03493.x	RCT (173)	Moderate to severe COPD. Outpatients from COPD clinic at a general Hospital in Northern Ireland	67 ± 7.9 55% female	SGRQ, COPD Knowledge, MAQ	Pharmacy-led disease and medicine management intervention vs. usual care Knowledge, understanding, communication Included Communication, Understand, and Use aspects of HL	**Medication Adherence, HRQL and Health resource utilization**	There were significant differences between the intervention and usual care groups regarding decreased in ED visits (p = 0.02), reduced hospitalizations (p = 0.01), increased adherence to medication (p = 0.019), and greater knowledge scores (p = 0.001). On the SGRQ scores, significant differences were reported in the intervention group on the symptom (p = 0.04) and impact subscales p = 0.03) but not on the physical activity subscale. The clinical pharmacy-led disease management program may improve adherence, reduce the need for hospital care in patients with COPD and improve aspects of their HRQL
Wilson SR, Strub P, Buist AS, Knowles SB, Lavori PW, Lapidus J, Vollmer WM. Shared treatment decision making improves adherence and outcomes in poorly controlled asthma. Am J Respir Crit Care Med. 2010;181(6):566–77. https://doi.org/10.1164/rccm.200906-0907OC	RCT (612)	Poorly or very poorly controlled Asthma Patients from five lung specialty clinical Sites in the US	46.9 ± 6 12 55.9% female	ATAQ, ATC, HRQL, MAQ and health care utilization	Shared Decision-making (SDM) vs. Clinician decision-making (CDM. Disease Knowledge, Included Communication, Understanding, and Use aspects of HL	**Medication Adherence, asthma control and HRQL**	Medication adherence was significantly higher in the SDM group (p < 0.0001) compared with usual care (CMA). The SDM group also had significantly better asthma control (p = 0.0225) than the CDM group. Asthma QOL improved significantly in both study groups over time, but did not differ significantly from each other. Asthma-related visits were significantly lower after the intervention in both study groups but did not differ significantly from each other. The SDM approach is efficacious in improving both medication adherence and clinical outcomes among poorly controlled asthma patients
Chavannesa N, Grijsen M, van den Akker M. et al. Integrated disease management improves one-year quality of life in primary care COPD patients: a controlled clinical trial. Prim Care Respir J. 2009; 18 (3): 171–176	RCT (162)	Mild to moderate COPD Patients form community primary care setting in Netherlands	63 ± 5 81	SGRQ,CCQ, HLQL, MMRC,MAQ. Healthcare utilization and self-efficacy	Integrated disease management (IDM) vs. standard care Included Understanding and Use aspects of HL	**Dyspnea, self-efficacy and HRQL**	Dyspnea rates decreased (p = 0.001) and QOL improved significantly in the intervention group (p = 0.002) compared to the usual care group. The IDM intervention improved quality of life in primary care COPD patients, compared to usual care. The improvement in SGRQ was both clinically relevant and statistically significant, in the intervention group

b							
Source	Design (Sample size)	Population/Disease type/Setting	Age/Sex	Tools applied to measure behavioral outcomes of interest	Intervention	Main outcome measured	Key findings & conclusions
Efraimsson EO, Hillervik C, Ehrenberg A. Effects of COPD selfcare management education at a nurse-led primary health care clinic. Scand J Caring Sci. 2008; 22(2): 178–185. DOI:10.1111/j. 1471–6712.2007.00510.x	RCT (52)	Moderate, severe or very severe COPD. Patients from Swedish primary care setting	68 ± 9.7 50% female	SGRQ, COPD Knowledge, smoking habits	Structured educational intervention vs. standard care Included Communication, Understanding, and Use aspects of HL	**Self-care, Smoking Cessation, Disease knowledge and HRQL**	A statistically significant increase was noted in the intervention group on SGRQ scores (p = 0.00035), dyspnea (p = 0.0267), quality of life ((p = 0.00030)), the number of patients who stopped smoking (p = 0.0185), and patients’ knowledge about COPD (p < 0.001) when compared to usual care group. The evidence from this study suggests integrating a structured program with self-care education to usual care can motivate patients’ lifestyle changes
Pur Ozyigit L, Ozcelik B, Ciloglu SO, Erkan F. The effectiveness of a pictorial asthma action plan for improving asthma control and the quality of life in illiterate women. J Asthma. 2014; 51:423–8. 10.3109/02770903.2013.863331	RCT (40)	Illiterate women with moderate-severe persistent asthma Three clinics in a under-developed province in Turkey	34.8 ± 6.88 100% female	ACT, HRQL SGRQ, and healthcare utilization	Educational intervention and access to pictorial asthma action plan vs. asthma education alone Included Understanding, and Use aspects of HL	**Asthma control and quality of life**	ACT and HLQOL scores of both groups improved significantly at the follow-up stage (p < 0.001), but did not differ significantly from each other (p < 0.07). The SGRQ scores at follow up were significantly higher than the control group (p = 0.033). The ED visits were reduced significantly in intervention group (p = 0.001). Use of pictorial asthma action plan in addition to patient education provides a significant improvement in asthma control, HRQL, and managing of asthma treatment in illiterate asthma women. These findings suggests that education and management plans for asthma patients from various socio-cultural levels should be tailored based on literacy and understanding level
Wan ES, Kantorowski A, Homsy D, Teylan M, Kadri R, Richardson CR, et al. Promoting physical activity in COPD: Insights from a randomized trial of a web-based intervention and pedometer use. Respiratory Medicine. 2017; 130: 102–110. https://doi.org/10.1016/j.rmed.2017.07.057	RCT (114)	Veteran COPD patients in 1 general pulmonary clinics in the US	68.6 ± 8.3 98.5% male	SGRQ, HRQL, MMRC, COPD Knowledge, and motivation and confidence to exercise and perform daily walking	Pedometer and website educational intervention vs. Pedometer alone Included Understanding, and Use aspects of HL	**Physical Activity, daily walking, self-efficacy, disease knowledge**	Daily step counts, 6-min walking, and physical activity increased over 3 months in the intervention group compared to control group (all p = 0.02). Self-efficacy and disease knowledge improved in both groups, but no significant differences were noted between groups. A website portal added to the pedometer use improved daily step counts, and sustained walking in COPD patients
Mendes de Oliveira, J.C., Studart Leitão Filho, F.S., Malosa Sampaio, L.M. et al. Outpatient vs. home-based pulmonary rehabilitation in COPD: a randomized controlled trial. Multidiscip Respir Med. 2010; 5(6): 401–408. https://doi.org/10.1186/2049-6958-5-6-401	RCT (117)	Clinically stable COPD patients. Inpatients and outpatients treated at the Institute of the Lung PR center in Brazil	69.2 ± 8.7 76.5% male	BODE Index^15^, MMRC and 6MWT scales	Supervised hospital-based vs. unsupervised home-based pulmonary rehabilitation Included Communication, Understanding, and Use aspects of HL	**Rehabilitation Program Attendance and Adherence**	There was a significant difference in the distance covered on the six-minute walk test and MMRC in both study groups after participating in the PR program (p < 0.05), but the groups did not differ significantly from each other (p = 0.44). There was a significant reduction in the BODE index in both study groups at the end of the study (p < 0.001). However, no significant difference was found between the outpatient and at-home groups (p = 0.90), Findings of this study demonstrates that a self-monitored home pulmonary rehabilitation program can achieve similar results to a supervised outpatient pulmonary rehabilitation program and is a valid alternative in the therapeutic approach to patients with COPD
Alsomali HJ, Vines DL, Stein BD, et al. Evaluating the effectiveness of written dry powder inhaler instructions and health literacy in subjects diagnosed With COPD. Respir Care. 2017;62(2): 172–178. https://doi.org/10.4187/respcare.04686	Educational RCT (24)	Stable COPD. Outpatients in pulmonary function laboratory of an urban teaching medical center in the US	65.6 ± 10.0 63% female	S-TOFHLA, Inhaler technique, ability to use peak flow meter, disease knowledge	Educational intervention vs. Usual care (control) Included Understanding, and Use aspects of HL	**Inhaler technique and ability to perform peak respiratory fellow**	The education improved patients’ inhaler technique (p < .002), but not the ability to perform proper peak flew meter use ((p = 0.96)). Health literacy was not associated with the ability to learn inhaler technique (p = 0.85). Use of inhaler device handouts alone without any verbal instructions or demonstration improved inhaler technique. Reading ability, as tested by the Health literacy tool, was not associated with patients’ capability to learn inhaler technique using written educational handout (p > . 0.05)
Janson SL, McGrath KW, Covington JK, Cheng S, Boushey HA. Individualized asthma self-management improves medication adherence and markers of asthma control. Journal of Allergy and Clinical Immunology. 2009; 123 (4): 840–846. https://doi.org/10.1016/j.jaci.2009.01.053	RCT (95)	Moderate-to-severe asthma. Outpatients from private and public community clinics in the US	38.3 ± 9.3 53% female	HLQOL, ACT, Peak flow, MAQ, and ICS adherence (assessed by the electronic device validated for monitoring metered dose inhaler use)	Computer-generated individualized self-management education intervention vs. self-monitoring alone process Included Understanding, and Use aspects of HL	**ICS adherence, HRQL, proper use of Peak inspiratory flow**	Mean ICS adherence improved in both study groups over time, but stayed consistently higher over time in the intervention group but the differences were not statistically significant (p = 0.79). The incidence of rescue medication use decreased significantly over time in both study group (p < .001), with no significant differences between groups. The mean change in symptom scores also decreased significantly for both groups over time, and the change rates were not significantly different between the two groups (p = 0.19). The peak flow scores improved significantly for both intervention and control groups during the intervention period, with no significant differences in the change rates between groups (p = 0.62). Perceived control of asthma and QOL improved significantly in the intervention group (p = 0.006, and p = 0.07), respectively. Intervention subjects reported significantly more changes in self-management behavior in the study group than in the control group (p < 0.0005). Individualized self-management education coupled with self-monitoring of asthma symptoms, and peak flow confers additional benefits in adults with asthma beyond self-monitoring alone
Poureslami I, Nimmon L, Doyle-Waters M, Rootman I, Schulzer M, Kuramoto L, et al. Effectiveness of educational interventions on asthma self-management in Punjabi and Chinese asthma patients: a randomized controlled trial. J Asthma. 2012;49:542–51. 10.3109/02770903.2012.682125	RCT (92)	Moderate to severe asthma. One specialty lung clinic in Canada	62.9 ± 15.3 50.6% female	Functional knowledge. Open-ended HL questionnaire, inhaler techniques (use a standardized nine-step observational checklist), MAQ, Medication adherence, Disease Knowledge	Culturally and linguistically sensitive educational intervention vs. routine patient education Included Communication, Understanding, and Use aspects of HL	**Asthma control, asthma knowledge, inhaler technique, HRQL**	Proper use of inhalers improved significantly in both study groups (p < 0. 001), with female participants showed significantly greater improvements compared to male patients (p = 0.04). Participants’ knowledge of asthma symptoms improved significantly over time in all participants (p < 0.01), with more improvements were observed among patients with high school diploma or more education (p = 0.03) and patients younger than 60 years (p < 0.01). Patients’ understanding of physicians’ instructions improved significantly over time among all participants (p < 0.01), with female participants showed significantly greater improvements compared to male patients (p < 0.05). Language and cultural barriers were identified as major issues to access and communicate with care providers. Access to culturally and linguistically appropriate educational material (written in community language and audio-visual instructions in story-format) improved disease-related knowledge, helped better understand and act on asthma symptoms, and promoted self-management practices among asthma patients from ethno-cultural communities
Wang KY, Chu NF, Lin SH, Chiang EC, Perng WC, Lai HR. Examining the causal model linking health literacy to health outcomes of asthma patients. Journal of Clinical Nursing. 2014; 23 (13–14): 2031–2042	Observational study (326)	Asthma. Pulmonary medicine outpatient departments at three medical centers and a regional teaching hospital in Taiwan	51 ± 18.3 50.7% female	TOFHLA^16^, asthma knowledge, inhaler techniques; healthcare use and PHQ	Assessed how low health literacy may influence health outcomes in adult asthma patients Checked Reading ability, Literacy, Understanding	**Health literacy skills and Engagement in Self-Management**	Overall, 72.3% of participants had adequate functional health literacy, based on TOFHLA scoring scale. Health literacy correlated positively with inhaler technique performance (p = 0.009), but correlated negatively with self-management behavior (p = 0.779). Health literacy correlated positively with asthma knowledge (p < 0.001), asthma attitudes (p = 0.001) and medical decision-making (p = 0.007) but correlated negatively with medical care experience (p = 0.639). Health literacy intervention can improve health out-comes of asthma patients. Increasing health literacy may lead to improved self-efficacy and control of asthma symptoms and help to reduce emergency department visits and hospitalizations
Press VG, Arora VM, Trela KC, et al. Effectiveness of Interventions to Teach Metered-Dose and Diskus Inhaler Techniques. A Randomized Trial. Ann Am Thorac Soc. 2016;13(6): 816–824	RCT (120)	Asthma and COPD. Inpatients at a teaching hospital in the US (82 Asthma and 38 COPD)	48.5 73% female	TOFHLA, Inhaler technique, MAQ, and Disease Knowledge	Comprehensive Teach-to-goal (TTG) Educational intervention vs. brief verbal Instruction (BVI) intervention Included Communication, Understanding, and Use aspects of HL	**Inhaler Technique and exacerbation**	Immediately after the interventions, the inhaler techniques significantly improved among the TTG group (p = , 0.001), but was not sustained at 30 days follow-up (p = 0.11). Acute care events were less common among teach-to-goal participants than brief intervention participants were at 30 days (p = 0.02), but not at 90 days (p = 0.6). Inpatient treatment-to-goal education may be an important first step toward improving self-management and health outcomes for hospitalized patients with asthma or COPD, especially among patients with lower levels of health literacy
Beatty CR, Flynn LA, Costello TJ. The Impact of Health Literacy Level on Inhaler Technique in Patients With Chronic Obstructive Pulmonary Disease. J Pharm Pract. 2017 Feb;30(1):25–30. https://doi.org/10.1177/0897190015585759. Epub 2016 Jul 10. PMID: 26033793	RCT (46)	Moderate to severe COPD. Patients in a community hospital in the US	67 ± 10.5 82.6% male	REALM-SF^17^, and Inhaler technique	Plain language handouts vs. standard hospital education materials Included Understanding, and Use aspects of HL	**Inhaler Technique and medication adherence**	Correct use of inhaler improved significantly among participants in both study groups, at the end of intervention, with significantly more improvements observed in intervention group compared to control group (p = 0.03). There was no significant difference in health literacy scores between the study groups at the end of the study (p > 0.05) There is a need for multiple educational modalities written in simple language for COPD patients, especially those with low health literacy to help them involve in disease management of their health condition

c							
Source	Design (Sample size)	Population/Disease type/Setting	Age/Sex	Tools applied to measure social and psychological outcomes of interest	Intervention	Main Outcome measured	Key findings & Conclusions
Apter AJ, Wan J, Reisine S, Bender B, Rand C, Bogen DK, et al. The association of health literacy with adherence and outcomes in moderate-severe asthma, Journal of Allergy and Clinical Immunology. 2013;132(2): 321–327, https://doi.org/10.1016/j.jaci.2013.02.014	RCT (284)	Moderate or severe asthma. Outpatients primary care and asthma from specialty practices of a inner-city hospital in the US	48 ± 14 71% female	S-TOFHLA, ANQ, HRQL, and ACT	Individualized problem-solving (PS) strategy vs. standard asthma education (AE) Included Communication, Understanding, and Use aspects of HL	**Adherence to ICS, HRQL and exacerbation rate**	Higher HL was significantly associated with better Asthma quality of life (p = .006), asthma control (p = .005), and medication adherence (NS) in both study groups at the end of the study period (6-month). There were no significant interaction between time and HL or between group assignment (PS, AE) and HL. In adults with moderate or severe asthma, higher health literacy scores were associated with better subsequent quality of life and asthma control. The relationship between HL and health is complex, and this study illustrated such complexity and pointed out that this is more than a cross-sectional association
Steurer-Stey C, Dalla Lana K, Braun J, Ter Riet G, Puhan MA. Effects of the “Living well with COPD” intervention in primary care: A comparative study. Eur. Respir. J. 2018, 51, 1701375	RCT (467)	Moderate to severe COPD. Patients from several primary care settings in Switzerland	67.7 ± 10.1 54.18% male	CRQ and PACIC^18^.HLQL, CCQ, CTCPSQ	Self-management intervention (LWWCOPD) vs. usual care **Checked Knowledge and Behavior change**	**Exacerbation, health related quality of life, and medication adherence**	The intervention group showed significant, clinically relevant improvement in all CRQ subscale scores (p < 0.05) and had considerably fewer moderate to severe exacerbations rates compared to the control group (NS). Significant increases were observed in intervention group patients’ confidence in performing the correct inhalation technique (p < 0.001) and confidence in the timely and correct use of the action plan (p < 0.05). No significant differences between the two groups were found related to smoking cessation and self-efficacy scores (NS). The structured self-management intervention program effectively improved disease coping skills and quality of life, and reduced the risk of exacerbations in patients with COPD
Thomas RM, Locke ER, Woo DM, Nguyen EHK, Press VG, Layouni TA, Trittschuh EH, Reiber GE, Fan VS. Inhaler Training Delivered by Internet-Based Home Video-conferencing Improves Technique and Quality of Life. Respir Care. 2017; 62(11):1412–1422. https://doi.org/10.4187/respcare.05445	RCT (48)	COPD. Parents in Veterans Affairs Health Care System in the US	67.5 ± 6.6 93% male	Hl (used 1-item question on self-reported confidence filling out medical forms), CRQ, HLQL, COPE^20^, and MAQ	Internet-based home videoconference educational intervention using teach-to-goal (TTG) approach vs. standard care Included Communication, Understanding, and Use aspects of HL	**Self-Esteem, inhaler technique, and COPD quality of life,**	COPD self-confidence (coping skills), inhaler adherence, and COPD quality of life improved significantly following the intervention (p < .0.003, (p < 0.045, and p < 0.001), respectively. Inhaler training using teach-to-goal methodology delivered by home videoconference is a promising means to provide training to patients with COPD that can improve technique, quality of life, self-efficacy, and adherence
Martin MA, Catrambone CD, Kee BA, Evans AT, Sharp LK, Lyttle C, et al. Improving asthma self-efficacy: Developing and testing a pilot community-based asthma intervention for African American adults, Journal of Allergy and Clinical Immunology, 2009; 123 (1): 153–159.e3, https://doi.org/10.1016/j.jaci.2008.10.057	RCT (107)	Poorly controlled persistent asthma. Patients from 2 primary care clinics in the US	37 ± 8 69.05% female	CES-D scales^19^, HRQL, COPE, ASES^21^, and PHQ	Face-to-face educational intervention vs. educational handouts Included Communication, Understanding, and Use aspects of HL	**Self-efficacy, disease management**	The intervention group had higher asthma self-efficacy (p < 0.001), use of asthma action plan (p = 0.06), higher HRQL (p = 0.002), and improved coping (p = 0.01) compared with the control group patients. Trends in behavioral and clinical outcomes favored the intervention group but were not statistically significant. A community-based asthma intervention improved asthma self-efficacy, self-perceived coping skills, and asthma quality of life for low-income adult patients
Poureslami I, Kwan S, Lam S, FitzGerald JM. Assessing the effect of culturally-specific educational interventions on attaining self-management skills for COPD in Mandarin and Cantonese speaking patients. Int J Chron Obstruct Pulmon Dis. 2016; 3 (11):1811–1822	RCT(91)	Moderate to severe COPD. Outpatients from ethno-cultural communities in one pulmonary medicine clinic Canada	60.23 ± 18.4 78.1% male	HL (too developed for HL and clinical outcomes assessment), Inhaler techniques, CRQ, SGRQ, and Disease Knowledge	Culturally, linguistically, and literally appropriate educational self-management Interventions vs. pictorial self-management Pamphlet Included Communication, Understanding, and Use aspects of HL	**Empowerment, self-efficacy, and COPD management**	Compared to the control subjects, patients in the Intervention group, had significantly more improvements in managing a COPD exacerbation (p < 0.01), better inhaler techniques (p < 0.001), ability to achieve goals in managing COPD (p < 0.01), and better understanding of pulmonary rehabilitation procedures (p < 0.05). Culturally appropriate educational interventions designed specifically to meet the needs of COPD patients are associated with better understanding of self-management and its practices
Paasche-Orlow MK, Riekert KA, Bilderback A, et al. Tailored education may reduce health literacy disparities in asthma self-management. Am J Respir Crit Care Med. 2005; 15: 172(8): 980–986	RCT (73)	Asthma. Inpatients in two inner-city hospitals in the US	40.9 ± 10.9 66% female	S-TOFHLA, ASES,MAQ, ATAQ, Inhaler techniques	Educational intervention vs. standard care Checked Literacy, and Understanding	**Improved knowledge, medication adherence**	After education, HL was positively associated with disease knowledge (p = 0.05) in all intervention patients. In addition, inhaler technique was significantly improved with the intervention patients with low HL (p = 0.02). Inadequate health literacy was not associated with poor adherence to corticosteroid therapy and Asthma Symptom Control (p = 0.86 and p = 0.84, respectively). Inadequate asthma self-management and HL skills are common. In this study, inadequate HL was associated with worse asthma medication knowledge and inhaler techniques, but it was not associated with medication adherence and asthma control

^π^ Randomized controlled trial = RCT; ^1^Short Test of Functional Health Literacy in Adults = S-TOFHLA; ^2^Asthma Control Test = ACT; ^3^Health Related Quality of life = HRQL; ^4^Health Literacy Survey-European Union (HLS-EU), ^5^Chronic Respiratory Disease Questionnaire (measures 4 domains (dyspnea, fatigue, emotional function, and mastery) of COPD-specific quality of life) = CRQ; ^6^Modified Medical Research Council Dyspnea Scale = MMRC; ^7^eHealthLiteracy Scale = eHEALS; ^8^Asthma Numeracy Questionnaire = ANQ; ^9^St George’s Respiratory Questionnaire = SGRQ; ^10^The six-minute walk test = 6MWT; ^11^COPD Transitional Care Patient Satisfaction Questionnaire = CTCPSQ’ ^12^Morisky adherence questionnaires = MAQ; ^13^Patient Health Questionnaire = PHQ; ^14^Clinical COPD Questionnaire = CCQ; ^15^Asthma Therapy Assessment Questionnaire = ATAQ; ^16^BODE Index = Body-mass index, airflow Obstruction, Dyspnea, and Exercise = BODE; ^17^Test of Functional Health Literacy in Adults = TOFHLA; ^18^Rapid Assessment of Adult Literacy in Medicine—Short Form = REALM-SF; ^19^Patient Assessment of Chronic Illness Care = PACIC; ^209^The Center for Epidemiological Studies-Depression (Assess Depressive symptoms) = CES-D; ^21^Coping Orientations to Problems Experienced Scale = COPE; ^22^Asthma Self-Efficacy Scale = ASES, Emergency visit = ED Visit

This recognition of the importance of HL has since led to development and testing of several HL measurement tools [47–49] for use in healthcare settings:

i. the Rapid Estimate of Adult Literacy in Medicine (REALM)—a word-recognition assessment [50];
ii. the Test of Functional Health Literacy in Adults (TOFHLA)—involving reading appointment slips, interpreting prescriptions, and filling in missing words on a consent form [51];
iii. the Wide Range Achievement Test (WRAT)—assessing reading recognition, spelling and basic math skills [52]; and
iv. the Newest Vital Sign (NVS)—assessing reading and numeracy skills through nutrition labels [53].

These tools are brief and relatively easy to administer [54, 55], and previous authors have demonstrated relationships between HL scores on these instruments and outcomes such as disease knowledge, health prevention behaviors, and quality of life, across population groups [56–60]. Accordingly, some have encouraged their use in practice [61, 62]. However, these tools have also been criticized [17, 48, 54, 55] for their focus on general literacy skills [27, 35] rather than skills that define a health literate individual, including navigation, comprehension, motivation and activation, and self-efficacy [16, 63]. In addition, these instruments were developed for the general population, rather than for specific disease groups (which may have disparate needs), and with little or no patient input [48, 64]. Accordingly, many have argued that these existing HL measurement tools have limited validity and applicability in real-world healthcare settings [26, 34, 65–67] and emphasized the need for tailored approaches to measuring HL in specific disease populations [28, 36–39]. Although various such function-based HL measures have since emerged [68–72], their use has not yet been reported in patients with AD. To address this, we brought together patients, HL researchers, and respiratory care clinicians to develop a new function-based HL measurement tool (using realistic case scenarios) exclusively for asthma and COPD patients [68, 73–77], which is currently being validated [78].

### Health literacy in respiratory care—an under-recognized problem

**Fig. 1:**
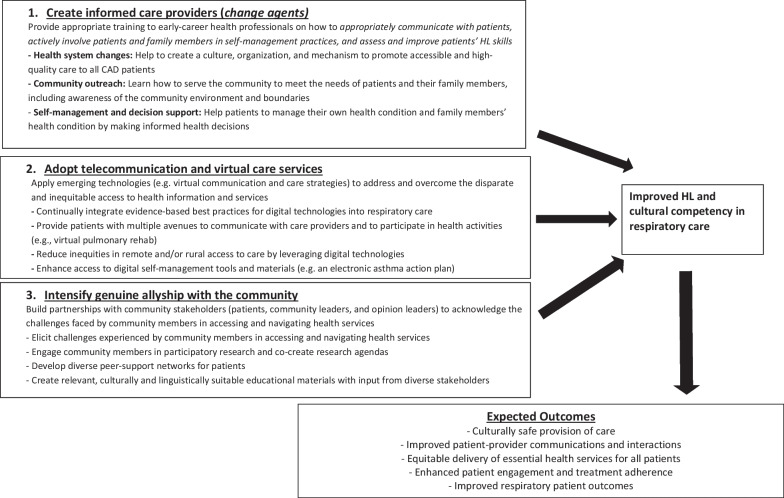
Three-level model strategies to promote health literacy and culturally competency in respiratory care

Asthma and COPD are among the most common chronic diseases, presenting a major and growing strain on global healthcare resources [21, 46, 79, 80]. Patients with these conditions should be empowered to act as informed decision-makers, develop partnerships with care providers, and self-manage their condition [3, 13, 81]. This requires a high degree of self-efficacy, achieved by obtaining and comprehending information and instructions about their health condition and its treatment [82–85]. However, patient engagement in such decision-making is dependent on the social determinants of health, including health beliefs and practices, attitudes, cultural norms, socio-economic status (SES), and baseline HL ([39–41, 86], Fig. 1).

Accordingly, providers can motivate and empower their patients to engage in disease management by improving their HL skills [83, 86–89]. The impact of improved HL skills could include slowing disease progression and improving patient-relevant health outcomes [74, 90–92]. Although respiratory organizations around the world have recognized the importance of addressing low HL [93–99] and several AD studies have administered HL measurement instruments, most of these tools focused merely on patient capabilities [8, 15, 16, 46, 100, 101], and were not specific to AD populations [16, 64, 72, 76, 100], thereby, limiting understanding of the impact of low HL on AD health outcomes [14, 15, 67, 102]. Prior investigators have suggested strategies to improve care for patients with low health literacy in clinical settings [5, 22, 37, 103] and some approaches have shown positive results in observational studies [104, 105], but, most of existing studies focus narrowly on educational interventions and corresponding outcomes related to comprehension, inhaler technique, and/or disease knowledge [106–109]. A previous systematic review [64] did not identify a single AD study that applied all five components of HL as part of an intervention. To gauge the existing state of interest and knowledge surrounding HL in AD, we sought to identify prior experimental and observational studies in ADs that assessed one or more specific component of HL (accessing, communicating, understanding, evaluating, and/or using information to improve disease outcome). These results are summarized in Table 2a–c, demonstrating the characteristics of each reviewed article.

Overall, we summarize 31 articles in this narrative review. None used a disease-specific HL assessment tool, and no single study applied more than three HL domains. The ‘understand’ aspect of HL and improving disease ‘knowledge’ (using knowledge questionnaires) were assessed in all 31 reviewed articles (100%). The ‘use’ domain of HL was identified in 25 articles (81%) of the articles. Use was simply assessed by directly assessing if participants applied the intervention in question (e.g., education) in managing their disease, improving medication adherence, and preventing exacerbations, or by indirectly assessing the impact of the intervention in improving the outcome of interest. ‘Communication’ was the least assessed HL domain, which was identified in only 17 (55%) of the reviewed articles, assessed by measuring the impact of communicating with a health care provider on outcomes of interest. The ‘numeracy’ domain was applied in only two studies (6%), which assessed understanding of numerical concepts such as dose change instructions for self-management of asthma or COPD. Lastly, ‘access’ and ‘evaluation’ domains of HL were each assessed by only one article (3%). Access was assessed by evaluating access barriers to healthcare services and relevant disease management education, and ‘evaluation’ was assessed by measuring patients’ ability to judge the severity of disease symptoms required to initiate needed treatment according to their action plan.

Even when HL was assessed, measurements in individual studies were limited to *associations* between baseline HL and trial outcomes (e.g., behavioural, healthcare services utilization, and health outcomes) among asthma and COPD patients [90–92]. No trial design attempted to improve HL skills through an intervention in order to measure the impact of changes in HL on patient or health system outcomes. For instance, in Azkan Ture et al. study, [110] inadequate HL was more common in patients with severe COPD than those with milder disease. Similarly, several studies demonstrated significant associations between HL and improved self- efficacy (Fan et al. [62]; Martin et al. [82]), and disease control (Wilson et al. [111]; Janson et al. [112]). Others reported correlations between baseline HL and quality of life (Goeman et al. [113]) and Thomas et al. [114]); medication adherence and use (Apter et al. [91]) and (Khdour et al. [115]); hospitalization (Wang et al. [116]), emergency department (ED) visits (Pur Ozyigit et al. [117]), and appropriate response to symptom worsening (Poureslami et al. [84]). Overall, findings consistently showed that patients with low HL skills had lower adherence to their medications and treatment plan, visited the ED more frequently, and had more asthma/COPD-related hospital admissions/re-admissions, and more symptom flare-ups than patients with higher HL skills. Studies also showed that HL was positively correlated with improved non-medical determinants of health. For instance, Eikelenboom et al. [118] found a link between HL levels and adopting healthier nutrition and having improved patient activation levels. Other researchers found significant associations between HL skills and exercise capacity (Wang et al. [116]), smoking cessation (Efraimsson et al. [119], and medical decision making (Wang et al. [90]. Despite these promising results, the mechanisms behind the reported associations between HL and respiratory outcomes remain unclear, as we did not identify any interventional studies that sought to enhance HL and measure impact on outcomes (e.g. inhaler technique, awareness and control of symptoms, management of acute exacerbation, and proper use of healthcare services). Accordingly, the causal relationship between HL and health outcomes requires further investigation [23, 101, 120].

### Patient HL challenges and potential respiratory care system responses

In the following section, we highlight challenges faced by patients with AD and low HL in actively engaging in disease management, and practice- and system-level changes required to address these barriers and drive improvements in HL.

#### Accessing health information and services

Limited access includes both the availability and attainability of information and services [121, 122]. Disadvantaged individuals experience inadequate access for several reasons [58, 104]; (1) they have less regular primary care visits [11, 118, 123]; (2) they are more prone to accessing healthcare information from unreliable sources outside of the medical system (e.g. a friend with a "similar" health condition, family members, neighbors, or the internet) [10, 23, 84, 102]; (3) even when referred to specialty clinics (including respiratory clinics), these settings are particularly poorly suited to offering culturally sensitive and/or same-language care to patients of diverse backgrounds [123–128].

Given that culturally matched patient-provider interactions have been shown to augment patient engagement in disease management and to improve health outcomes [125, 126, 129, 130], healthcare systems must invest in improving competencies and diversity of personnel (language and cultural) in order to render all care services attainable to all members of the community [2, 30, 102, 105]. This can be supplemented by provision of multi-lingual health information (written and/or electronic) that is also easily understandable and relevant across ethnicities and cultures.

#### Processing and understanding information and instructions

Respiratory care providers often overestimate patients’ HL skills, assuming that complex instructions have been understood [32, 132]. This issue is compounded by the fact that many patients with limited HL also overestimate their own ability to process and understand medical instructions [61, 111, 133]. In addition to verbal communication, printed disease-related educational materials are often inaccessible to low HL patients due to an inappropriately high reading grade requirement for comprehension [23, 93, 97, 128, 134] (low HL and low literacy and reading skills are closely associated [8, 9, 135, 136]).

To address these issues, both care and accompanying educational materials must be tailored to the diverse needs and abilities of patients across different ethnic and cultural communities, ages, and socioeconomic classes [90, 137, 138]. A suggested approach to foster open, interactive patient-provider communication is to compliment plain language resources [56, 132, 139, 140] with a “teach back” approach (asking the patient to repeat back what was understood), to ensure that patients have understood information correctly [141, 142], stimulating dialogue and question-asking [143]. This approach has been shown to improve medication adherence and inhaler technique in AD patients [133, 144, 145]. Patient input in material development can help to ensure that reading levels and content are properly matched to the target audience, and optimize both content and layout, thereby enhancing understanding and uptake [118, 137, 144, 146]. Specifically, incorporation of patient input in self-management tools for asthma and COPD augments self-management behaviors and improves outcomes, particularly in older patients [92, 113, 145, 147].

#### Appraising the quality of information and care services

To optimize health outcomes, AD patients must assess the quality and credibility of health information they encounter, and its relevance to their personal health needs [23, 87, 148].

Lacking corresponding critical appraisal and evaluation skills has emerged as a central issue in HL research in recent years [149, 150], but has barely been studied in respiratory research [66, 87, 149]. Accurate measurement of evaluation skills could help to identify the differences between patients’ expectations and their perceptions of the services and information received [61]. This evaluation skill component of HL is understudied, particularly in AD [68, 78].

#### Applying information to make health-related decisions

Most attention in HL research has been focused on information availability, accessibility, and comprehension (readiness, attainability, readability, and comprehensibility of health-related information) [48, 58, 63]. However, maintaining health requires a series of practical acts, and obtaining and understanding relevant information does not equate to using it [114, 151]. Although all aspects of HL are important, the effectiveness of health information and services in *changing behavior* is what ultimately determines impact [17, 25]. Many patients with airways diseases have high levels of knowledge about their health condition [23, 120, 137, 148], but struggle to apply that knowledge in the disease management process [11, 12, 27, 107, 125, 135]. A person’s behaviours are also influenced by internal and external motivations, as well as their ability, readiness, and willingness to use the information received from care providers [19, 60, 102, 140, 147]. Additionally, factors such as beliefs and worldviews, the perceived trustworthiness and practicability/relevance of the information, and previous experiences all effects a person’s intention to apply the information [77, 78, 92, 152]. Accordingly, patient-provider interactions must go beyond information “transfer”, to facilitate behavior change [133, 140]. Improving patient educational materials to include personalized instructions (both related to the behaviour itself and how to achieve the behaviour change) may empower patients with the skills needed to change [90, 117, 139, 153]. Additionally, when appropriate, providers may augment this process by having patients *practice* relevant actions and procedures (and offer feedback) to compliment and reinforce verbal and written information [56, 132, 154].

### A model for health literacy and culturally competent respiratory care

Both cultural and social factors deeply influence the way people access and navigate health information and services [2, 41, 127, 155]. Culturally competent care systems understand and respect the health beliefs and practices of their patients, appreciate language barriers, and apply such understanding in practice [126, 134, 156–158]. Accordingly, HL competent care facilitates equity of essential healthcare services for all community members [14, 37–41, 103]. Increasing diversity in healthcare providers themselves (including in leadership and governance) and use of patient navigators (trained health workers) to assist vulnerable patients with language and/or literacy barriers may help to address system inequities [102, 159]. However, implementation of these strategies may be hindered by various countries’ population structures (i.e. a lack of sufficient representatives to play these roles across diverse cultural and language groups) [134, 160].

#### A three-level model

Patients with AD, particularly older patients and COPD patients are among population groups with the lowest HL levels [92, 145, 147]. To achieve the goal of creating a responsive, patient-centered system of care for AD patients, we propose new strategies, in a three-level model format (Fig. 1), with special focus on training and empowering healthcare professionals to excel in roles as *change agents* for bridging cultural and HL gaps in their own patients [161]. The change agents can also leverage rapidly accelerating virtual care and communication technologies to address inequitable access to health information and care; and the last is to broaden the healthcare services team by building partnerships with (culturally competent) community stakeholders [162, 163]. We applied the three-level model strategies in our recent research projects [10, 15, 68, 84, 125, 128, 130, 146, 164, 165]. The results of our studies demonstrated potential efficacy of the proposed strategies and the need for further prospective validation. These strategies and their expected outcomes are outlined below.

Firstly, respiratory clinics should train providers to recognize the heterogeneity in patients’ beliefs, preferences, limits, and needs, and consider these in their communication style and clinical practices [102, 157, 162, 163]. There is evidence that improved provider communication skills and awareness of social determinants of health mitigate impacts of limited HL and cultural mismatch [86, 157, 166]. With appropriate training (in university for future health professionals and through ongoing/continued education for current staff), respiratory health professionals can acquire the skills required to act as change agents [167], by engaging in patient questions, explaining treatment instructions while avoiding medical jargon, and using strategies such as the teach-back method [108, 140, 166]. The focus of a change agent is to improve a patient's capacity and motivation to engage in self-management (one of the foundational components of AD management). Given the impact of social determinants of health [161], this role may extend beyond medical practice to a global assessment and support of financial and social factors impacting adherence, motivation, and treatment response [102, 168]. This approach has been shown to improve self-management and outcomes in this population [3, 88, 89, 92, 145, 154]. However, a sustainable model will require advocacy regarding the importance of non-medical determinants of health in respiratory disease management [169] to ensure that these aspects are included in future curricula and programming, and receive sufficient funding.

Secondly, to address disparate and inequitable access to health information and services, respiratory care providers must espouse emerging technology, in the form of *virtual communication and care strategies*. The goal is to overcome care access barriers related specifically to patients living in remote or rural areas and/or having difficulties securing time away from work for appointments during normal office hours [121, 164, 170–173]. With technological advancements as well as increased provider and patient acceptance of and access to remote communication models driven by the COVID-19 pandemic [174, 175] (even among lower socioeconomic class groups), telehealth can now be used to address essential healthcare services across patient populations [173]. It can also facilitate health education for patients and communication between primary care physicians and specialists [176, 177]. Although telehealth-based interventions improved knowledge [176], emotional and mental health [173], quality of life [172], medication adherence [178], hospitalization and emergency department (ED) visits [179], and self-monitoring [176, 178] across chronic diseases, there are no high-quality studies evaluating this in AD [177]. Ideas such as an electronically accessible action plan with weekly text message reminders to assess one’s asthma control [165], and virtual pulmonary rehabilitation (PR) (telerehab) [172, 180] hold promise [181]. For example, a telerehab program can provide educational materials online, with the patient attending practical sessions (e.g., exercise, breathing/cough control training) via interactive video conferencing [180, 181]. Such a program was shown to improve exercise capacity, health related quality of life, and psychological status [180, 181]. This approach also enables access for those living in remote locations and whose physical limitations and/or capacity to secure transit impairs in-person attendance [180]. In fact, virtual care is often favored by patients and providers alike due to convenience and flexibility [174].

Finally, a successful model must build *partnerships with community stakeholders* (patients, community leaders, and opinion leaders). Partnerships lead to allyship—through insights into the challenges faced by community members in accessing and navigating health services [82, 118]. This can occur as part of community care, or, for example, community AD patients might be involved in participatory research (from the beginning of the research process) and/or in developing educational material [82, 118, 131, 164]. For example, we successfully gauged AD patients’ research priorities through a series of focus groups across Canada [10, 73, 84, 146, 182] and applied these in the Canadian Respiratory Research Network’s research prioritization exercise (https://respiratoryresearchnetwork.ca/). We also engaged patients, community healthcare providers, and clinicians in developing audio-visual educational materials on AD topics in seven different languages [125, 128, 130, 163, 183]. This work enabled us to establish a peer-support network [127] that offers newly diagnosed patients with AD the opportunity to gain insights from those with lived experience in managing AD [184, 185]. These groups also provided an opportunity for individuals of diverse cultural backgrounds and HL levels to interact with others in a familiar language and at a comparable level of sophistication. Such peer support and patient networks have been shown to reduce patients’ feelings of isolation and fear, to enhance their mental capacity to cope with their condition, and to build the confidence needed to engage in self-management [132, 184–186]. Care system-community collaboration has also been shown to facilitate delivery of effective education to disadvantaged patients [1, 11, 68, 111, 185, 187, 188].

## Conclusions

As patients with AD are increasingly expected to actively engage in disease self-management, we must acknowledge the responsibility of the health system to ensure that they have the capacity to execute such complex tasks, by addressing their HL [30, 37, 41, 105, 171]. A respiratory care system that reinforces HL in a culturally competent way will improve health outcomes through patient engagement, clearer communication, and improved patient-provider interactions. Key components of system change include training healthcare providers to become change agents, accelerating adoption of evidence-based virtual communication and care strategies, and building partnerships with community stakeholders. These changes will reduce socio-cultural and socio-economic disparities in care access and quality, yielding enormous benefits for patient outcomes, possibly with reductions in healthcare costs [46].

There are exciting research opportunities to design and evaluate novel strategies to both measure HL and to address cultural competency and HL in patients with AD. Longitudinal research is particularly needed to evaluate which health outcomes are improved by addressing HL in a culturally competent way, including the sustainability of observed effects. As communication technologies continually advance, research is also needed to determine the most efficient and effective strategies to enable virtual care. Ultimately, our common goal should be to realize a patient-centered respiratory care system that engages willing patients not only in decision-making around their own care, but also in the development of the very educational material that is presented to them and the very research, which establishes their therapy.

## Data Availability

The authors have made readily reproducible materials described in the manuscript, including the software used, databases and all relevant raw data, and made them freely available to any scientist wishing to use them, without breaching participant confidentiality**.**

## Abbreviations

HL: Health literacy
COPD: Chronic obstructive pulmonary disease
AD: Airway disease
PR: Pulmonary rehabilitation
